# Assessing the accuracy of electronic health record gender identity and REaL data at an academic medical center

**DOI:** 10.1186/s12913-023-09825-6

**Published:** 2023-08-22

**Authors:** Rachael Proumen, Hannah Connolly, Nadia Alexandra Debick, Rachel Hopkins

**Affiliations:** 1 Department of Medicine, State University of New York (SUNY) Upstate Medical University, 750 E. Adams St, Syracuse, New York USA; 2 State University of New York (SUNY) Upstate Medical University Norton College of Medicine, Syracuse, New York USA; 3 Department of Medicine, Division of Endocrinology, State University of New York (SUNY) Upstate Medical University, 750 E Adams St., Syracuse, NY 13210 USA

**Keywords:** Electronic health record, Race and ethnicity, Language, Gender identity, Data quality

## Abstract

**Background:**

Collection of accurate patient race, ethnicity, preferred language (REaL) and gender identity in the electronic health record (EHR) is essential for equitable and inclusive care. Misidentification of these factors limits quality measurement of health outcomes in at-risk populations. Therefore, the aim of our study was to assess the accuracy of REaL and gender identity data at our institution.

**Methods:**

A survey was administered to 117 random patients, selected from prior day admissions at a large academic medical center in urban central New York. Patients (or guardians) self-reported REaL and gender identity data, selecting from current EHR options. Variables were coded for the presence or absence of a difference from data recorded in the EHR.

**Results:**

Race was misreported in the EHR for 13% of patients and ethnicity for 6%. For most White and Black patients, race was concordant. However, self-identified data for all multiracial patients were discordant with the EHR. Most Non-Hispanic patients had ethnicity correctly documented. Some Hispanic patients were misidentified. There was a significant association between reporting both a race and an ethnicity which differed from the EHR on chi square analysis (P < 0.001). Of those who reported an alternative ethnicity, 71.4% also reported an alternative race. Gender identity was missing for most patients and 11% of the gender-identity entries present in the EHR were discordant with the patient’s self-identity. Preferred language was 100% concordant with the EHR.

**Conclusions:**

At an academic medical center, multiracial and Hispanic patients were more likely to have their demographics misreported in the EHR, and gender identity data were largely missing. Healthcare systems need strategies that support accurate collection of patients’ self-reported ReAL and gender identity data to improve the future ability to identify and address healthcare disparities.

**Supplementary Information:**

The online version contains supplementary material available at 10.1186/s12913-023-09825-6.

## Introduction

Collection of accurate patient demographics including race, ethnicity, preferred language (ReAL) and gender identity in the electronic health record (EHR) is important for achieving equitable and inclusive care. A foundation of accurate ReAL data can allow healthcare organizations to perform quality improvement studies and identify groups at risk for poor outcomes or inadvertently receiving lesser care [1]. Collection of REaL data is mandated by the Centers for Medicare and Medicaid Services in the United States and recommended to be captured in the EHR [2, 3]. As of 2023, Federally Qualified Health Centers require collection of sexual orientation and gender identity data for those at least 18 years of age [4].

Organizations are unable to accurately assess healthcare disparities if REaL and gender identity data are inaccurate or missing [5]. Therefore, an important first step in these efforts is to assess the accuracy of these data in the EHR. However, misidentification of race and ethnicity is a recognized problem in the quality of social determinants of health (SDoH) data [6, 7]. Accurate collection of gender-identity data is even more challenging. Since 2014, the Affordable Care Act’s Meaningful Use program has required that Medicaid provider EHRs be able to collect gender identity data. However, for many reasons, this information is often missing [8].

Given these challenges, the aim of our study was to assess the accuracy of REaL and gender identity data recorded in the EHR at an urban academic medical center by determining the extent to which a patient’s race, ethnicity or gender identity predict whether those data are correctly entered into an EHR?

## Methods

A paper survey was administered to adult and pediatric patients (n = 117) during admission to an academic medical center from February 3rd, 2022 to March 31st, 2022. The study was performed at a not-for-profit, 752-bed teaching hospital with a level-1 trauma center, a comprehensive stroke center, and the region’s only children’s hospital and cancer center. The medical center is located in a small city and serves an extensive geographic region encompassing urban, suburban, and rural areas of central New York State in the United States. The city in which the medical center is located hosts a large refugee population with several different languages and dialects. The EHR used in this study was EPIC Systems.

At our institution, patients’ demographic information is obtained by a clerk during the registration process for both inpatient admissions and outpatient visits. The clerk then enters this information directly into the EHR. This process requires that the clerk feels comfortable enough to ask the patient for specific information and remembers to do so and requires that the patient is willing and able to give an accurate answer.

To select patients for the survey, patients with odd-numbered medical record numbers were selected from EHR-generated lists of admissions from the prior day. Medical students, who received training prior to data collection, were responsible for survey administration, including reading a scripted dialog to the patients explaining the process. Patients (or guardians) were given the opportunity to self-report their race, ethnicity, preferred language, and gender identity by selecting from available options in our EHR, or by selecting ‘other’ and writing in their response (appendix 1). Available race selections included the major categories as documented by the Office of Management and Budget (OMB) (White, Black, American Indian/Alaska Native (AIAN), Asian and Pacific Islander) as well as more granular selections (see appendix 1, question 2) [9, 10]. Ethnicity options included initially choosing between Hispanic or Non-Hispanic, as suggested by the OMB, as well as over 45 selections for those who identify as Hispanic, in addition to ‘unknown’ and ‘another Hispanic, Latino/a or Spanish origin’.

Participants were directed to complete the survey as best they could, and the data collectors were directed to only provide aid if requested. Patients were excluded if survey administrators or patients were unavailable at the time of survey collection. Patients were also excluded if they were under enhanced airborne precautions. The accuracy of each demographic variable was determined by agreement of the results between the patients’ self-reported survey responses and what was documented in the EHR at the conclusion of the survey collection period. Each demographic variable was coded for the presence or absence of a difference from the value listed in the EHR. For patients with multiple admissions during the study period, only the initial encounter was recorded. Patients with discrepancies between self-reported and EHR data were given the choice to have their EHR updated. These changes were made in the EHR after study results were analyzed. This study was deemed exempt from review by the State University of New York (SUNY) Upstate Institutional Review Board.

For data analysis, individuals who selected two or more races were considered multiracial. For individuals who reported an ethnicity as ‘other’ with a response that was discordant from the EHR, this was considered as a change from the EHR.

Statistical analyses were conducted using IBM SPSS Version 28.0. Bivariate testing for categorical variables was done via chi square. In the accompanying tables, we only depict the discordance from the EHR for White, Black, American Indian/Alaskan native (AIAN) and multiple race persons as the other categories were too small to analyze or not represented in our sample. We used logistic regression to evaluate the effects of patients’ race and ethnicity on the accuracy of those data in the EHR. Primary diagnosis and insurance type were also considered; however, the data was too heterogenous to draw conclusions and thus were not included. A p-value of 0.05 was used to determine statistical significance. For regression analysis, race was dichotomized into White, and Non-White due to the small sample size of AIAN, Other and multiracial responses in this sample. Ethnicity was also dichotomized into non-Hispanic and Hispanic due to the small sample size of patients that did not identify as non-Hispanic or Hispanic.

We evaluated to what extent a patient’s race predicts whether race is correctly entered into the EHR. To address this aim, Eq. 1 estimates the odds of adjusting race in the EHR based on dichotomized race.

$${\text{Equation 1: ln}}{\left({\frac{{\hat y}}{{1 - \hat y}}} \right)}{\text{ = }}{{\text{b}}_{\text{0}}}{\text{ + }}{{\text{b}}_{\text{1}}}{\text{White }}$$

Equation 2 accounts for the relationship between the odds of adjusting race and race, controlling for ethnicity using a logistic regression.

$$\begin{gathered}{\text{Equation 2: ln}}{\left({\frac{{\hat y}}{{1 - \hat y}}} \right)}{\text{ = }}{{\text{b}}_{\text{0}}}{\text{ + }}{{\text{b}}_{\text{1}}}{\text{White }} \hfill \\\,\,\,\,\,\,\,\,\,\,\,\,\,\,\,\,\,\,\,\,\,\,\,\,\,\,\,\,\,\,\,\,\,\,\,\,\,\,\,\,\,\,\,\,\,\,\,\,\,\,\,\,\,\,\,\,\,\,{\text{ + }}{{\text{b}}_{\text{2}}}{\text{Non-Hispanic}} \hfill \\ \end{gathered}$$

Next, we sought to evaluate to what extent a patient’s ethnicity predicts whether it is correctly entered into an EHR. To address the second aim, Eq. 3 estimates the odds of adjusting ethnicity in the EHR based on dichotomized ethnicity.

$${\text{Equation 3: ln}}{\left({\frac{{\hat y}}{{1 - \hat y}}} \right)}{\text{ = }}{{\text{b}}_{\text{0}}}{\text{ + }}{{\text{b}}_{\text{1}}}{\text{Non-Hispanic}}$$

Equation 4 accounts for the relationship between the odds of adjusting ethnicity and ethnicity, controlling for race using a logistic regression.

$$\begin{gathered}{\text{Equation 4: ln}}{\left({\frac{{\hat y}}{{1 - \hat y}}} \right)}{\text{ = }}{{\text{b}}_{\text{0}}}{\text{ + }}{{\text{b}}_{\text{1}}}{\text{Non-Hispanic }} \hfill \\\,\,\,\,\,\,\,\,\,\,\,\,\,\,\,\,\,\,\,\,\,\,\,\,\,\,\,\,\,\,\,\,\,\,\,\,\,\,\,\,\,\,\,\,\,\,\,\,\,\,\,\,\,\,\,\,\,\,{\text{ + }}{{\text{b}}_{\text{2}}}{\text{White}} \hfill \\ \end{gathered}$$

## Results

241 potential patients were identified using the process described above, of which 117 completed surveys. 124 patients were excluded. 123 were excluded because either the patient was not available when the survey administrator attempted to speak with them, or the survey administrators were unavailable. One patient declined to participate. The survey was conducted in English. None of the approached patients requested or required language interpreter services, which were available for all patients through electronic tablets.

The distribution of race in our sample after correction of misreported race was as follows: White 78.6%, Black 13.4%, AIAN 0.9%, and multiracial 7.1%. Ethnicity was distributed as 93.1% non-Hispanic, 5.1% Puerto Rican, 0.9% Columbian, and 0.9% other. Overall, self-reported race was discordant with the EHR for 13% of patients, and ethnicity 6%, respectively. Patients were more likely to self-report multiple races as compared to the data in the EHR. Race was correctly documented in the EHR for the majority of White and Black patients. However, all multiracial patients were incorrectly entered in the EHR. Of four patients with race listed as ‘other’ in the EHR, half chose a specific race (1 White, 1 Black), and half selected ‘unknown’. Most non-Hispanic patients had ethnicity correctly documented. Some Hispanic patients were misidentified (5/7 Puerto Rican patients, 1 patient who identified as Columbian, 1 who identified as multi-ethnic). The rate of missing responses for race was 3%. The distribution of gender identity in our sample was 47.0% cis female, 47.9% cis male, 0.9% transgender female, 0.9% transgender male, and 3.4% gender neutral.

Gender identity was absent in the EHR for most patients. Of those initially documented, 11% were found to be discordant with the patient’s self-identity. Preferred language (English) was concordant with the EHR in 100% of patients (Table 1).

**Table 1:** Sample Characteristics of EHR data as compared to Self-reported survey data

	EHR data		Self-reported data	
	Frequency	Percent	Frequency	Percent
Race ^a^				
White	97	82.9	88	78.6
Black	15	12.8	15	13.4
American Indian or Alaskan Native	1	.8	1	.9
Multiracial	4	3.4	8	7.1
Ethnicity				
Not Hispanic or Latino	110	94.0	109	93.1
Puerto Rican	2	1.7	6	5.1
Columbian	0	0.0	1	.9
Nicaraguan	1	.9	0	0.0
Other	3	2.6	1	.9
Gender Identity				
Not Documented	100	85.5		
Cis Female	8	6.8	55	47.0
Cis Male	8	6.8	56	47.9
Transgender Female	0	0.0	1	0.9
Transgender Male	1	0.9	1	0.9
Gender Neutral	0	0.0	4	3.4
Preferred Language				
English	117	100.0	117	100.0
Other	0	0.0	0	0.0
	**Frequency**		**Percent**	
Race Corrected in EHR				
Yes	15		13.0	
No	110		87.0	
Ethnicity Corrected in EHR				
Yes	7		6.0	
No	110		94.0	
Gender Identity Corrected in the EHR ^b^				
Yes	2		11.8	
No	15		88.2	
Preferred Language Corrected in the EHR				
Yes	0		0.0	
No	117		100.0	

^a^ Within our sample, 5 patients did not confirm their self-reported race

^b^ Most surveyed patients did not initially have a gender identity documented in their EHR. If they did, and it was corrected, it is represented here

On chi square analysis, there was a significant association between reporting both a race and an ethnicity other than what was documented in the EHR (P < 0.001). Of those who reported an alternative ethnicity, 71.4% also reported an alternative race (Table 2).

**Table 2:** Chi-Square Analysis of Demographic Variables: Race and Ethnicity

	Change in Ethnicity					
	No		Yes		χ ^2^	p-value
Change in Race						
No	98 (90.7)		2 (28.6)		22.4	< 0.001
Yes	10 (9.3)		5 (71.4)			

Odds ratios (ORs) were estimated from logistic regression models predicting the odds of patients adjusting their self-reported race as compared to the value in the EHR (Table 3). As shown in Model 1 in Table 3, race is a statistically significant predictor of patients’ self-reported race being discordant with the current value in the EHR. The odds of an incorrect race being listed in the EHR for white patients was approximately one-quarter the odds of an incorrect race being listed in the EHR for non-White patients. However, controlling for ethnicity, the odds of adjusting race were no longer statistically significant.

**Table 3:** Estimated Odds Ratios from a Logistic Regression, Predicting Race Adjustment in EHR by Race. Notes: Model 2 was adjusted for ethnicity. *p < 0.05, **p < 0.01, ***p < 0.001

	Model 1	Model 2
White	0.24* (0.08, 0.79)	0.71 (0.14, 3.63)
Non- Hispanic		0.03** (0.002, 0.30)

Odds ratios were also estimated from logistic regression models predicting the odds of patients having a different self-reported ethnicity from the value in the EHR (Table 4). As shown in Model 1 in Table 4, ethnicity is a statistically significant predictor of patients’ self-reported ethnicity being discordant with the current value in the EHR. Compared to Hispanic patients, non-Hispanic patients had approximately 99% decrease in odds of incorrectly recorded ethnicity. Controlling for race, the odds of incorrectly recorded ethnicity for non-Hispanic patients remained statistically significant.

**Table 4:** Estimated Odds Ratios from a Logistic Regression Predicting Ethnicity Adjustment in EHR by Ethnicity. Notes: Model 2 was adjusted for race. *p < 0.05, **p < 0.01, ***p < 0.001

	Model 1	Model 2
Non- Hispanic	0.01*** (0.001, 0.06)	0.01*** (0.001, 0.11)
White		0.75 (0.06, 9.82)

## Discussion

Many sociodemographic factors including race, ethnicity, language, gender, social status and culture, can have a significant impact on one’s health [11]. There is growing recognition and interest in the impact of these social determinants of health.

We found that both race (for multiracial patients) and ethnicity (for Hispanic patients) were more likely to be misreported in our EHR and that there was a statistically significant association between a difference in both race and ethnicity from what was documented in the EHR. According to the 2022 National Healthcare Quality and Disparities report, 10.4% of patients self-identify as two or more races, a number which has increased steadily over the last several years [12]. Our EHR system overall misrepresented our patients’ race 13% of the time, similar to previous findings at a Veteran’s Affairs Medical Center, which found self-reported race and ethnicity was discordant with the EHR 15.7% of the time [3, 13].

The EHR used in this study, EPIC systems, allows those who identify as multiracial the ability to select multiple races in the EHR, as recommended by the OMB [10]. However, in our sample, we found that those individuals who identify as multiracial were not identified as such in the EHR. This could possibly be due to our small sample size, or the result of some registration clerks being unaware of the ability to select multiple races in the EHR. Alternatively, during registration patients could be selecting only one race due to societal pressures of selecting the “most applicable choice”. Our findings are consistent with prior studies showing that multiracial or Hispanic patients are more likely to have inaccuracies in their documented race and ethnicity [6, 14–16]. This is an important issue to address as the number of individuals who identify as multiracial is rapidly increasing [4, 10].

The process for obtaining REaL and gender identity data is fraught with challenges including varying definitions of race, patient suspicion, patient privacy concerns, and inadequate staff training [1, 8, 9]. Hospital staff, such as registration clerks, may feel uncomfortable collecting this mandated data, due to lack of guidance on the purpose of collecting the data, what happens to the data after collection and with whom it is shared [17]. While in most health care organizations the registration clerk is the entity responsible for obtaining these demographics, patients may feel more comfortable having this information obtained by their nurse or physician [18]. In many institutions, including ours, the process for collecting these demographic data is underdeveloped.

We expected some variation in preferred language in our survey results, however, all respondents chose English as their primary language, which did not differ from the EHR. This is consistent with previous data on self-reported preferred language, which found a concordance rate of 95% with the EHR and is likely due to legally mandated reporting of preferred language in the EHR [2]. Additionally, patients’ primary language and preferred language may differ in the healthcare setting, for ease of interaction with providers and staff.

It is known that when seeking healthcare, transgender individuals, among other gender minorities, report high levels of discrimination and barriers to care, including lack of relevant provider training [11, 20]. Gender identity was not documented for most patients in our sample. A 2020 study of 49,314 individuals admitted at Rush University Medical Center found that 76% (37,371) had missing gender identity data, consistent with a prior report in 2019 ^8^. Further analysis of our data shows that when comparing patients’ gender identity to legal sex in the EHR, there were 7 discrepancies and 6 were patients who self-identified as gender-neutral or transgender. This suggests that gender identity is likely being recorded primarily for transgender or gender-neutral individuals. Normalizing collection and documentation of self-identified gender can contribute to better health outcomes and allow a better understanding of health disparities in gender minority patients [19, 20].

The gold standard for collection of REaL data and gender identity is through patient self-reporting, given the complexities of collection [1, 2, 6, 10]. An improved registration process would include informing patients of the reason behind data collection. It has been noted that when patients are informed of the reason behind the collection of this sensitive information, they are much more comfortable, especially when the reason is for achieving a high quality of care [15, 18]. This improved process would also allow informed patients to self-report their demographics in a universal, easy-to-use format which could potentially reduce errors and improve the completeness and quality of EHR data.

Our study has limitations. The study was performed at a single institution, and had a relatively small sample size, which may limit generalizability. EHR information was input by registration clerks at each inpatient or outpatient visit. Therefore, data from outpatient visits may possibly be included in this data set. Additionally, we do not know the exact circumstances for which each patients’ data was initially entered into the EHR. There is a possibility that some patients came in emergently, or were very ill, and thus the accuracy of their data may have been affected. We are also unable to account for whether there was a difference in severity of illness between included and excluded patients. Given that many patients were excluded because they were unavailable for procedures or imaging, they may well have been a more seriously ill population which might have introduced some inadvertent bias. Minors’ responses are reflective of their guardian’s survey completion and thus may not reflect individuals’ true responses. Some respondents provided multiple responses or ‘other’, likely due to the fluidity and variability in definitions of race and cultural influences. Finally, the social implications of inquiring about these factors may have caused response bias.

Nonetheless, our study results reveal that discrepancies persist between self-reported REaL and gender identity data and that documented in the EHR. Standardizing improvements in the collection of REaL and gender identity data in the EHR would help ensure accurate patient demographics which are essential for identifying and addressing healthcare disparities.

### Electronic supplementary material

Below is the link to the electronic supplementary material.

Supplementary Material 1

## Data Availability

The datasets used and/or analyzed during the current study are not publicly available due to patient privacy but are available from the corresponding author on reasonable request.
