# Supplementary material for: Assessing the accuracy of electronic health record gender identity and REaL data at an academic medical center
Source: BMC Health Serv Res. 2023 Aug 22;23:884. doi: 10.1186/s12913-023-09825-6 (PMC10463428; doi:10.1186/s12913-023-09825-6)
Supplement: Supplementary file 1 — Supplementary Material 1 [file 12913_2023_9825_MOESM1_ESM.docx]

**Appendices:**

**Appendix 1: Survey.**

Assessing the Accuracy of Self-Identified Patient Demographics in EPIC

The purpose of this survey is to see if the demographic information (race, ethnicity, preferred language, and gender identity) entered in our record system at the time of hospital admission is accurate.

Sometimes, for many reasons, this data is collected incorrectly or not at all. Our hope is that by verifying that we have the correct information about our patients, we will be able to understand and improve our ability to provide equitable and inclusive care to all patients.

The person presenting this survey to you is not directly involved in your medical care and your willingness to participate in this project will not in any way impact the care you receive at Upstate University Hospital. Also, your answers to this survey will not in any way impact the care you receive at Upstate University Hospital.

The survey will be de-identified and your data will not be shared.

If you are willing to participate, please answer the following questions:

1. Are you of Hispanic, Latino, or Spanish Origin?

If **NO,** please SKIP to **question 2.**

If **YES,** from the choices below, please select which best describes your **ethnicity.**

| 𝤿Andalusian | 𝤿 Canal Zone | 𝤿 Central American Indian | 𝤿 Criollo | 𝤿Guatemalan | 𝤿 Mexican American | 𝤿 Panamanian | 𝤿Salvadoran |
| --- | --- | --- | --- | --- | --- | --- | --- |
| 𝤿 Another Hispanic, Latino/a, or Spanish origin | 𝤿 Canarian | 𝤿 Chicano | 𝤿 Cuban | 𝤿Honduran | 𝤿 Mexican American Indian | 𝤿 Paraguayan | 𝤿South American |
| 𝤿Argentinean | 𝤿 Castillian | 𝤿 Chilean | 𝤿 Dominican | 𝤿 La Raza | 𝤿 Mexicano | 𝤿 Patient Refused | 𝤿South American Indian |
| 𝤿Asturian | 𝤿Catalonian | 𝤿 Colombian | 𝤿 Ecuadorian | 𝤿 Latin American | 𝤿 Nicaraguan | 𝤿 Peruvian | 𝤿Spaniard |
| 𝤿 Belearic Islander | 𝤿 Central American | 𝤿 Costa Rican | 𝤿 Gallego | 𝤿 Mexican | 𝤿 Not Hispanic | 𝤿 Puerto Rican | 𝤿 Spanish Basque |
| 𝤿 Unknown | 𝤿 Uraguayan | 𝤿 Valencian | 𝤿 Venezuelan | **Other:** (Fill in here) |  |  |  |

1. From the choices below, please select which best describes your **race**.

| Patient Refused | 𝤿 American Indian or Alaska Native | 𝤿 𝤿 Asian Indian | 𝤿 Bangladeshi | 𝤿 𝤿 Bhutanese | Black or African American | 𝤿 Burmese | 𝤿 Cambodian |
| --- | --- | --- | --- | --- | --- | --- | --- |
| Carolinian | 𝤿 Canarian | 𝤿 Chamorro | 𝤿 𝤿Chinese | Chuukese | Fijian | 𝤿 Filipino | 𝤿 Guamanian |
| 𝤿 Hmong | 𝤿 Indonesian | 𝤿 Iwo Jiman | 𝤿 Japanese | 𝤿 𝤿 Kiribati | 𝤿 Korean | 𝤿 Kosraean | Laotian |
| 𝤿 Madagascar | 𝤿 Malaysian | 𝤿 Maldivian | 𝤿 𝤿 Mariana Islander | 𝤿 Marshallese | Melanesian | 𝤿 𝤿 Native Hawaiian | Nepalese |
| 𝤿 New Hebrides | Okinawan | 𝤿 𝤿 Other Asian | Other Pacific Islander | 𝤿 𝤿 Pakistani | 𝤿 Palauan | 𝤿 Papua New Guinean | 𝤿 Pohnpeian |
| 𝤿 Saipanese | 𝤿 Samoan | 𝤿Singaporean | 𝤿Solomon Islander | 𝤿 Sri lankan | 𝤿 Tahitian | 𝤿 Taiwanese | Thai |
| Tokeleuan | Tongan | Unknown | 𝤿 Vietnamese | White or Caucasian | Yapese | **Other:** (Fill in here) |  |

1. From the choices below, please select which best describes your **preferred language**.

| Acholi | 𝤿 Afrikaans | Albanian | 𝤿 Amharic | Arabic | 𝤿 Arabic/Massalit | 𝤿 Arabic/Moroccan | Armenian |
| --- | --- | --- | --- | --- | --- | --- | --- |
| Bambara | 𝤿 Bengali | 𝤿 Bhutanese | Bislama | Bosnian | Bulgarian | Burmese | 𝤿 Cambodian/Khmer |
| Cantonese Chinese (inc Toishanese) | 𝤿 Chin/Burmese | 𝤿 Chin/Hindi | 𝤿 Chinese | Creole | 𝤿Croatian | Czech | 𝤿 Dari |
| Dinka | 𝤿 Diula | Dutch | English | Ewe | Farsi | 𝤿 Farsi (persian) | 𝤿 Fijan |
| French | 𝤿 𝤿 Fulani (Fula) | 𝤿 German | 𝤿 Grebo | Greek | 𝤿 Greenlandic/Kalaallisut | 𝤿 Gujarati | 𝤿 Haitian Creole |
| Hausa | 𝤿 Hebrew | 𝤿 Hindi | 𝤿 Hmong | Hungarian | 𝤿 𝤿 Indonesian | 𝤿talian | 𝤿 𝤿 Japanese |
| Kanjobal | 𝤿 Kannada | Karen | 𝤿 Karenni | 𝤿Kinyarwanda; Rwanda | 𝤿 Kirundi | 𝤿 Kizigua | Korean |
| 𝤿 Krahn/Kru/Sapo | Kurdish | Kurmanji | 𝤿 Laos | 𝤿 Latvian | Lingala | 𝤿 Lithuanian | 𝤿 Luo |
| 𝤿 Maay Maay | Macedonian | Malayalam | Mandarin Chinese | 𝤿 Mandingo | 𝤿 Nepali | 𝤿 Nuer | 𝤿 Oromo |
| 𝤿 Pashto | 𝤿 Pennsylvania Dutch | 𝤿 Persian | 𝤿 Polish | 𝤿 Portuguese | 𝤿 Pulaar | 𝤿Punjabi | 𝤿 Quechua |
| Rohingya | 𝤿 Romanian | 𝤿 Rundi/Burundi | 𝤿 Russian | 𝤿 Samoa | 𝤿 Sango | 𝤿 Serb/Croatian | 𝤿 Sign Language/American |
| 𝤿 Sign Language/Bhutanese | 𝤿 Sign Language/Burmese | 𝤿 Sign Language/Nepali | 𝤿 Sign Language/Somali | 𝤿 Sign Language/Tactile | Slovak | Somali | Soninke |
| Sorani/Iraqi | 𝤿 Spanish | 𝤿 Sudanese | 𝤿 Swahili; Kiswahili | 𝤿 Swedish | 𝤿 Tagalog | Tagalog/Phillipino | 𝤿 Taiwanese |
| Tamil | 𝤿 Telugo | 𝤿Thai | 𝤿 Tibetan | Tigrinya | Tongani | Turkish | 𝤿 Twi/Akan |
| 𝤿 Ukranian | Unknown | 𝤿Urdu | 𝤿 Urdo Pakistani | 𝤿 Vietnamese | 𝤿 Wolof | 𝤿 Wu Chinese (Shanghainese) | 𝤿 Yemeni Arabic |
| 𝤿 Yiddish | **Other:** (Fill in here) |  |  |  |  |  |  |

1. From the choices below, please select which best describes your **gender identity.**

| 𝤿 Female (cis) | 𝤿 Gender Neutral (i.e non-binary, ungendered, gender fluid, gender queer) | 𝤿 Transgender Female/Male-to-Female (assigned female at birth, “Trans Male”) |
| --- | --- | --- |
| 𝤿 Male (cis) | 𝤿 Other | 𝤿 Transgender Male/ Female-to-Male (assigned male at birth, “Trans female”) |

5. If we find the demographic information (race, ethnicity, preferred language, and gender identity) entered in our electronic medical record system is INCORRECT, would you like us to update it?

| YES | NO |
| --- | --- |
